# Salidroside enhances 5-fluorouracil sensitivity against hepatocellular carcinoma via YIPF5-induced mitophagy

**DOI:** 10.3389/fphar.2024.1503490

**Published:** 2025-01-06

**Authors:** Sumin Sun, Haili Hu, Feiyu Li, Sheng Huan, Long Chen, Jiahui Chen, Peihua Sun, Xiaoqing Dong

**Affiliations:** ^1^ College of Life Sciences, Joint Institute of Nanjing Drum Tower Hospital for Life and Health, Nanjing Normal University, Nanjing, China; ^2^ Department of Hematology, Nanjing Drum Tower Hospital Clinical College of Nanjing University of Chinese Medicine, Nanjing, China

**Keywords:** salidroside, 5-fluorouracil, mitophagy, senescence, mitosis, hepatocellular carcinoma

## Abstract

Hepatocellular carcinoma (HCC) is a major medical challenge due to its high incidence and poor prognosis. 5-Fluorouracil (5-FU), although extensively studied in the treatment of HCC and other solid tumors, has limited application as a first-line therapy for HCC due to its resistance and significant inter-patient variability. To address these issues, researchers have explored drug repurposing. One of our key findings in this endeavour was the potent anti-HCC effect of the natural product Salidroside (Sal) when co-administered with 5-FU. Sal was found to inhibit mitosis and promote cellular senescence in HCC cells via a mechanism distinct from 5-FU, specifically by inducing excessive mitophagy that led to cellular mitochondrial dysfunction. Importantly, YIPF5 was confirmed as a potential molecular target of Sal. This natural product modulated YIPF5-induced mitophagy and influenced both mitosis and senescence in HCC cells. The combination of Sal and 5-FU demonstrated significant therapeutic effects in a mouse HCC model. In conclusion, our study was not only in line with the innovative strategy of drug repurposing, but also important for drug design and natural product screening targeting the relevant pathways.

## 1 Introduction

HCC is an aggressive and swiftly advancing malignancy, accounting for a significant proportion of cancer-related fatalities on a global scale (Sung et al., 2021). The scarcity of noticeable early symptoms and the absence of robust diagnostic biomarkers often result in patients being diagnosed at intermediate to advanced stages (Ganesan and Kulik, 2023). Patients with advanced HCC often face the issue of resistance to chemotherapy and radiotherapy, greatly limiting their treatment options. In 2007, Sorafenib, as the first approved systemic therapy for advanced HCC, brought a new option with tyrosine kinase inhibitors for patients. In 2018, Lenvatinib became the second drug approved for first-line treatment of advanced HCC, followed by drugs such as Regorafenib, Cabozantinib, and Ramucirumab as second-line treatment options. In 2020, the immunotherapy combination of Atezolizumab and Bevacizumab marked the beginning of a new era, providing fresh insights into the combination of immunotherapies with other treatments (Llovet et al., 2022; Wu et al., 2021). Nevertheless, the therapeutic efficacy for HCC remains relatively constrained, underscoring the pressing need to identify novel drug regimens to combat this aggressive cancer.

5-FU is an important antimetabolite and cell cycle-targeting agent that has been widely studied in HCC and other solid tumors (Guo et al., 2021; Pranzini et al., 2022). However, the therapeutic window of 5-FU is narrow. In clinical treatment, most patients do not achieve optimal drug exposure, and only 20%–30% of patients have therapeutic concentrations within the effective range (Kaldate et al., 2012; Sethy and Kundu, 2021). According to the BCLC guidelines, 5-FU is not currently used in the clinical treatment of HCC (Reig et al., 2022). Drug repurposing includes the use of existing drug knowledge, clinical data, and bioinformatics tools to discover new drug indications, an approach that can significantly reduce drug development time, cost, and risk. The combination of drugs is an effective strategy for drug repurposing. Chinese medicine monomers, known for their unique biological activities and fewer side effects, are considered potential candidates for improving the sensitivity of chemotherapeutic drugs (Miao et al., 2023; Yu et al., 2023). Combining traditional Chinese medicine monomers with chemotherapeutic agents for the treatment of HCC may represent a new idea and direction in oncology.

The results of our bioinformatics analyses showed that the survival of HCC patients was highly correlated with the level of mitophagy. The PINK1/Parkin pathway is the most extensively studied mitophagy pathway (Li J. et al., 2023). When the mitochondrial membrane potential is impaired, the entry of PINK1 into the inner membrane of the mitochondria is blocked. Consequently, PINK1, which has accumulated in the outer membrane, recruits the E3 ubiquitin ligase Parkin. Parkin is activated by the phosphorylation of PINK1. Once activated, Parkin ubiquitinates mitochondrial outer membrane proteins following mitochondrial damage, recruiting downstream receptor proteins and initiating ubiquitin-dependent mitophagy (Han et al., 2023; Lu et al., 2023). Typically, researchers believe that mitophagy promotes tumour progression by degrading damaged mitochondria and reducing oxidative stress in tumour cells (Lin et al., 2023). However, mitophagy may also precede mitochondrial dysfunction. Studies have shown that excessive mitophagy can trigger mitochondrial dysfunction. Inhibition of mitophagy has been found to attenuate ketoconazole-induced mitochondrial dysfunction and apoptosis, suggesting that the activation of mitophagy in tumour cells could be an important strategy for attacking liver cancer (Chen et al., 2019). Tapping into low-toxicity mitophagy activators may be an effective new strategy to improve the sensitivity of clinical HCC chemotherapy.

Sal, the most effective physiologically active substance in Rhodiola rosea extracts, has been shown to play a role in enhancing PINK1/Parkin-mediated mitophagy in many diseases (Li and Chen, 2019; Zhang et al., 2018; Gu et al., 2020). Thus, we proposed that Sal may be a potential drug for chemosensitization in HCC, with this effect being produced by inducing mitophagy. In this study, we validated the efficacy of Sal in combination with 5-FU against HCC, which further lays the foundation for the optimization of the use of clinical chemotherapeutic agents.

## 2 Materials and methods

### 2.1 Reagents and antibodies

5-Fluorouracil (C_4_H_3_FN_2_O_2_) (F6627) was bought from Sigma-Aldrich (St Louis, MO, USA). Salidroside (C_14_H_20_O_7_) (HY-N0109) was purchased from MCE (New Jersey, USA). Dulbecco’s modified essential medium (DMEM) (319-000-CL), Roswell Park Memorial Institute 1640 (RPMI 1640) (350-000-CL), fetal bovine serum (FBS) (086–150), and trypsin–EDTA (325-043-CL) were obtained from WISENT (Saint-Jean-Baptiste, QC, CAN). Sodium Pyruvate (PB180422) was purchased from Procell (Wuhan, China). Primary antibodies against LC3 (14600-1-AP), Cyclin B1 (28603-1-AP), and YIPF5 (12931-1-AP) were purchased from Proteintech Group (Rosemont, IL, USA). The main antibodies against PINK1 (A11435), Parkin (A0968), CDKN2A/p16^INK4a^ (A11337), CDKN1A/p21 (A19094) and CDK1 (A12414) were obtained by ABclonal Biotechnology Co., Ltd (Wuhan, China).

### 2.2 Animal procedures and treatments

All animal experimental procedures were approved by the Institutional Animal Care and Use Committee of Nanjing Normal University (IACUC-20230911) and were conducted in compliance with the guidelines set forth by the Guide for the Care and Use of Laboratory Animals. Male BALB/c mice, aged 6–8 weeks, were purchased from the Jiangsu Huachuang Sino Pharmaceutical Technology Corporation (Taizhou, China). To construct an orthotopic HCC mouse model, H22 cells were administered into the BALB/c mice at a dosage of 1.5 × 10^^6^ cells in 1.8 mL and injected into the tail vein at high pressure within a span of 5–8 s (Li et al., 2011). A total of thirty-two mice were randomly assigned to four groups using a computerized randomization system. They were subsequently treated with the respective therapeutic agents and control solutions. Group 1 served as the control group and received phosphate-buffered saline (PBS). They were given 0.1 mL of PBS orally every 2 days and received an intraperitoneal injection of 0.1 mL PBS. Group 2 received Sal at a dosage of 100 mg/kg via oral gavage every 2 days. Group 3 was administered 5-FU at a dosage of 10 mg/kg via intraperitoneal injection every 2 days. Group 4 underwent a combined treatment of 100 mg/kg Sal via oral gavage and 10 mg/kg 5-FU via intraperitoneal injection, administered every 2 days. The treatment period for all groups lasted 4 weeks. Upon study completion, all mice were euthanised under anaesthesia with pentobarbital at a dosage of 50 mg/kg. Blood samples were collected for biochemical parameter assessment. The livers were excised, fixed in a 4% paraformaldehyde (PFA) solution, and embedded in paraffin for histopathological examination. The remaining liver tissues were used for protein extraction and analysis.

### 2.3 Serum biochemistry

Blood samples were carefully collected from the mouse orbit using clot-activating tubes, followed by centrifugation at 3000 RPM for 15 min to isolate the supernatant. Automatic biochemical analyzer (Hitachi, Tokyo, Japan) was employed to ascertain the levels of liver injury-related indicators (alanine aminotransferase (ALT) and aspartate aminotransferase (AST)) in mouse serum.

### 2.4 Histological analysis

Fresh liver cancer tissues were initially preserved in a 4% PFA solution for a duration of 12–24 h. Subsequently, they underwent a dehydration process using a graded series of ethanol concentrations, followed by a dialysis step with xylene. The tissues were then meticulously embedded in paraffin, sectioned, and allowed to dry. Hematoxylin-eosin (HE) staining was conducted as required to examine the pathological alterations within the liver tissue. Representative images were captured in a blinded and randomized manner from various fields of view.

### 2.5 Cell culture

The immortalised HCC cell lines Hepa1-6, HepG2, AML12 and 293T were purchased from the American Type Culture Collection (ATCC) (Manassas, VA, USA). Additional cell lines, H22 and Huh7, were provided by Procell Sciences Co., Ltd (Wuhan, China). Huh7 and HepG2 cells were cultured in DMEM supplemented with 10% FBS. The H22 cells were nurtured in RPMI 1640 medium, also enriched with 10% FBS. Hepa1-6 cells were specifically cultured in DMEM that included 1% sodium pyruvate and 10% FBS. AML12 cells were grown in a DMEM/F12, enriched with 0.5% Insulin-Transferrin-Selenium (ITS-G), 40 ng/mL dexamethasone, and 10% FBS. All cell cultures were maintained at a temperature of 37 °C within a humidified incubator, which was calibrated to contain an atmosphere of 5% CO_2_ to ensure optimal growth conditions.

### 2.6 Trypan blue staining

HCC cells in the logarithmic phase of growth were seeded at a density of approximately 30,000 cells per well in 24-well plates that were pre-coated with coverslips. After culturing the cells to adhere, HCC cells were treated with a specific concentration of Sal and 5-FU. Post-treatment, the cells were further cultured for 24 h. Subsequently, they were incubated with trypan blue (KeyGEN, Jiangsu, China) for 1–3 min. Following this, the coverslips were removed, and the cells were photographed to document the cell death.

### 2.7 Cell viability assay

Based on the previous literature (Huang et al., 2023a), the CCK8 assay was performed to investigate the viability of HCC cells. The absorbance values at 450 nm were measured using a Bio-Rad iMark plate reader.

### 2.8 Lentiviral shRNA knockdown

Stable Huh7 and Hepa1-6 cell lines expressing Pink1 shRNA, YIPF5 shRNA or non-targeting control shRNA were created using the pLKO.1-based lentiviral shRNA technique. The shRNA and packaging plasmids were transfected into 293T cells to produce the lentiviral particles that infected the target cell lines. The stable integration of the shRNA constructs was maintained by puromycin selection. The shRNA sequences are shown as follow: Human-Pink1 shRNA-1: 5′-CGGACGCTGTTCCTCGTTATG-3′; Human-Pink1 shRNA-2: 5′-CGG​CTG​GAG​GAG​TAT​CTG​ATA-3′; Mouse-Pink1 shRNA-1: 5′-GCG​GTA​ATT​GAC​TAC​AGC​AAA-3′; Mouse-Pink1 shRNA-2: 5′-CCT​GGC​TGA​CTA​TCC​TGA​TAT-3′; Human-YIPF5 shRNA-1: 5′-GCA​ATG​AAA​GTA​AAC​GTG​TAT-3′; Human-YIPF5 shRNA-2: 5′-AGTATGCTGGCTATGACTATT-3′; Mouse-YIPF5 shRNA-1: 5′-GCCATTCTATGGAGACAGCTT-3′; Mouse-YIPF5 shRNA-2: 5′-CCC​AAT​TAA​CTC​TGC​TGT​GAA-3′.

### 2.9 Real-time PCR

The total RNA was extracted, and the qPCR was performed using ChamQ SYBR qPCR Master Mix (Vazyme, Nanjing, China). The primers for qPCR are as follows: Mouse-Actb forward: 5′-CAT​TGC​TGA​CAG​GAT​GCA​GAA​GG-3′, Mouse-Actb reverse: 5′-TGC​TGG​AAG​GTG​GAC​AGT​GAG​G-3’; Mouse-CDK1 forward: 5′-CAT​GGA​CCT​CAA​GAA​GTA​CCT​GG-3′, Mouse-CDK1 reverse: 5′-CAAGTCTCTGTGAAGAACTCGCC-3’; Mouse-Cyclin B1 forward: 5′-AGAGGTGGAACTTGCTGAGCCT-3′, Mouse-Cyclin B1 reverse: 5′-GCA​CAT​CCA​GAT​GTT​TCC​ATC​GG-3’. Actb levels were taken for normalization.

### 2.10 Western blot analysis

After exposing HCC cells to various concentrations of Sal and 5-FU for 24 h, the proteins were extracted. The subsequent Western blot procedure was carried out, following the previous literature (Huang et al., 2023b). Antibodies were used according to the instructions and were configured at a dilution ratio of 1:1000. The intensity of the protein bands was quantified utilizing the ImageJ software.

### 2.11 Mitochondrial membrane potential detection

Changes in the mitochondrial membrane potential of HCC cells treated with Sal were evaluated using a mitochondrial membrane potential assay kit (JC-1) (Beyotime, Shanghai, China). Briefly, HCC cells were seeded at a density of 1 × 10^^5^ cells per well in 24-well plates and allowed to adhere to the plate walls. Following this, the cells were stained with JC-1 for 20 min at 37°C. After staining, the cells were washed twice with PBS to remove any unbound probes. The red and green fluorescence intensities, indicative of the mitochondrial membrane potential, were then measured using a fluorescence microscope (Leica, Wetzlar, Germany). Representative images of these fluorescence signals were captured for further analysis.

### 2.12 Transmission electron microscope

The medium from the HCC cell plates was discarded, and electron microscopy fixative (2.5% glutaraldehyde) was swiftly introduced. Cells were then scraped off and transferred to a centrifuge tube. After the cell precipitation was collected by centrifugation, the initial fixative was discarded. The cells were subsequently immersed in fresh fixative for 2 h, after which they were stored at 4°C for a minimum duration of 4 h. Post-glutaraldehyde fixation, the samples underwent rinsing and were further subjected to osmium tetroxide fixation for an additional 2 h. Following this, the samples were dehydrated through a graded series of ethanol concentrations. The subsequent steps included infiltration, embedding, and sectioning. The sections were then subjected to a double staining procedure using uranyl acetate and lead citrate for 15 min at room temperature. After staining, the sections were allowed to air dry overnight. The morphological, dimensional, and numerical alterations in the mitochondria of HCC cells, consequent to the intervention with Sal, were examined using TEM (FEI, Hillsboro, Oregon, USA).

### 2.13 Plate clone formation assay

HCC cells were plated at 500 cells/well in 6-well plates, treated with respective drugs, and cultured for 14 days. Subsequently, they were fixed with 4% PFA, stained using Giemsa stain (Leagene, Beijing, China), rinsed extensively with PBS, and once dried, the cells were photographed.

### 2.14 Wound healing

In the wound healing assay, HCC cells were seeded into 6-well plates and allowed to adhere overnight. The cell monolayer was then wounded by scratching it perpendicularly with a 200 μL sterile pipette tip. Detached cells were gently washed away with PBS, and the culture medium was replenished with one containing a reduced FBS concentration serum concentration of 2%. The wound area was monitored under a microscope, and images were captured. Following this, the cells were exposed to the designated drugs, returned to the incubator for 24 h, and then re-examined and photographed under the same microscopic field to assess the healing progress.

### 2.15 Senescence associated β-galactosidase (SA-β-Gal) activity

SA-β-Gal activity was assessed using the Senescence β-Galactosidase Staining Kit (Beyotime, Shanghai, China). The procedure was executed according to the manufacturer’s protocol.

### 2.16 Consensus clustering analysis of mitophagy

We accessed a dataset from The Cancer Genome Atlas (TCGA) that encompassed gene expression profiles, clinical details, and survival metrics for 374 HCC cases and 50 samples of adjacent or normal liver tissue. Following the exclusion of samples due to insufficient sequencing data, suboptimal expression data quality, incomplete data regarding adjacent or normal liver tissues, and missing follow-up information, the study ultimately encompassed 368 HCC patients. Consensus clustering analysis identified mitophagy subtypes in HCC. An unsupervised algorithm with Euclidean distance and Ward linkage was used, with the optimal cluster number determined by ConsensusClusterPlus in the R package, validated through 1000 repetitions. Principal component analysis assessed sample distribution among clusters. The clinical relevance was evaluated by comparing mitophagy subtypes with clinicopathological features and using the Kaplan-Meier method to analyze survival impact. Statistical significance was set at *p* < 0.05. The ggalluvial package visualized the relationship between clustering and clinical variables.

### 2.17 Statistical analysis

All results were expressed as mean ± standard deviation (SD) using the GraphPad Prism 8 (GraphPad software version 8.0). Statistical analyses were conducted using Student’s t-test for comparing two groups and one-way analysis of variance with post hoc Tukey’s test for comparing multiple groups. Individual cell experiments and animal experiments were performed in duplicate or triplicate and repeated 3 times using matched controls, and the data were pooled. In all analyses, the values of *p* < 0.05 were considered to indicate statistical significance.

## 3 Results

### 3.1 Salidroside enhances the sensitivity of 5-fluorouracil against HCC

**FIGURE 1:**
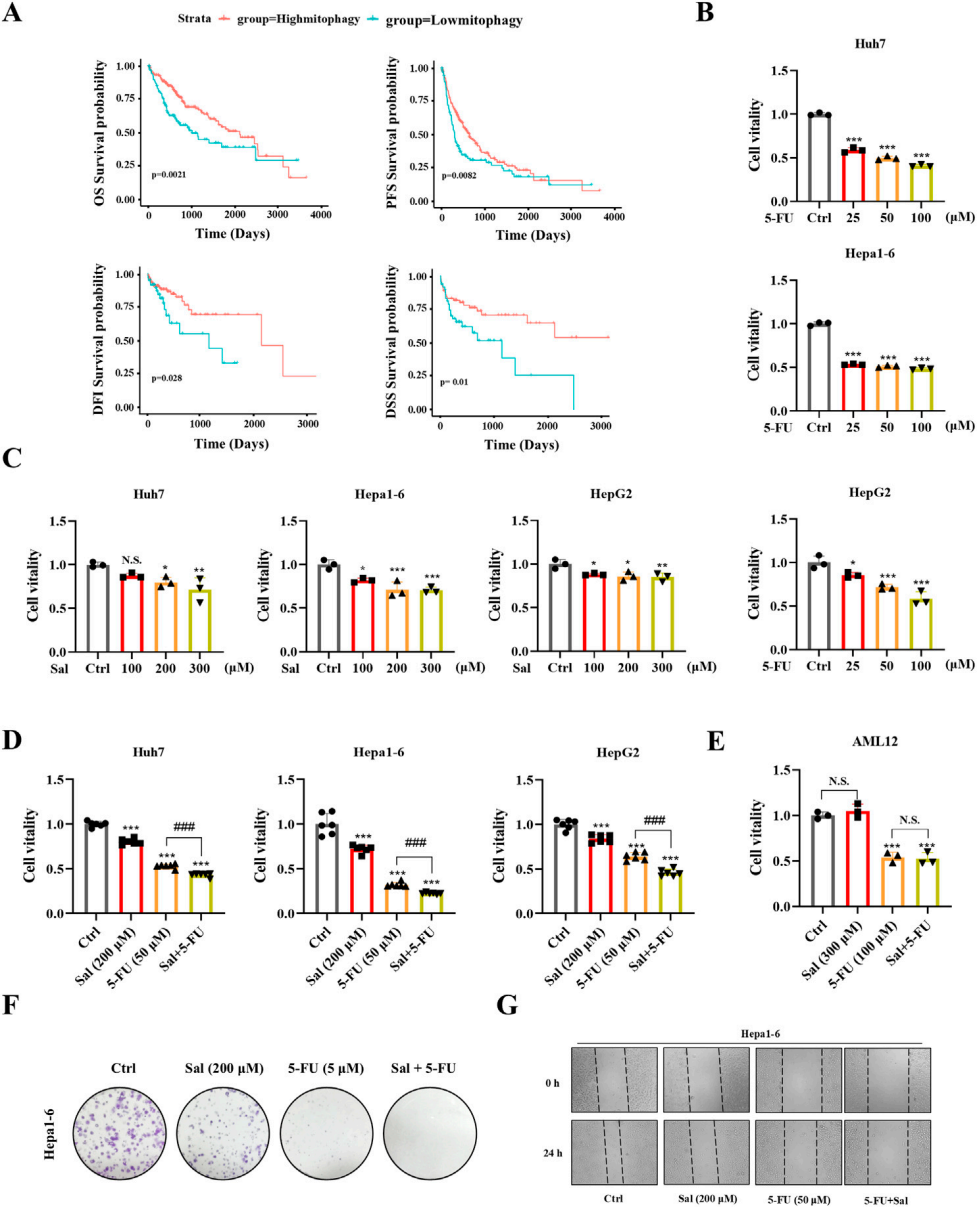
Salidroside enhances the sensitivity of 5-fluorouracil against HCC. **(A)** Kaplan-Meier curves for OS, DFI, DSS and PFS in patients with high and low mitophagy. **(B–D)** HCC cells were incubated with the indicated concentrations of Sal and 5-FU for 24 h, while the control group was treated with the control solvent. Cell viability was assayed by CCK8. **(E)** Mouse hepatocyte AML12 cell viability was assayed by CCK8. **(F)** The ability of Hepa1-6 HCC cells to form colonies under the treatment of Sal and 5-FU. **(G)** Wound healing assays were performed to detect Hepa1-6 cells migration. The data are expressed as mean ± SD (n = 3); *p < 0.05, **p < 0.01, and ***p < 0.001 vs. Control solvent. ^###^ *p* < 0.001 vs. 5-FU. N.S., not significant.

To delve into the genomic traits of mitophagy in HCC, the expression profiles of mitophagy genes from The Cancer Genome Atlas (TCGA) were analyzed. Employing unsupervised clustering, two distinct regulatory patterns were discerned: cluster 1, comprising 119 cases (low mitophagy level), and cluster 2, encompassing 249 cases (high mitophagy level) (Supplementary Figure S1A). Principal component analysis (PCA) corroborated these findings, revealing two distinct patient clusters (Supplementary Figure S1B). When assessing the single-sample gene set variation analysis (ssGSVA) scores for mitophagy, cluster 2 outperformed cluster 1 (Supplementary Figure S1C). In the TCGA cohort, patients with high levels of mitophagy had a better overall survival (OS), progression-free survival (PFS), disease-free interval (DFI) and disease-specific survival (DSS) survival prognosis than those with low levels of mitophagy (log-rank test, *p* ≤ 0.05) (Figure 1A). The findings implied that mitophagy could have positively influenced HCC, and increasing its levels might have enhanced drug responsiveness. Combining multiple drugs is a prevalent strategy to mitigate chemotherapy resistance (Yu et al., 2020; Li et al., 2021). Traditional Chinese medicine has a long-standing history of being utilized in the management of tumours, and it has demonstrated remarkable efficacy (Gao et al., 2016; Tan et al., 2023). Chinese herbs, known for their multi-target effects, low toxicity, and holistic approach, could be synergistically used with 5-FU. Sal has been reported to be an effective mitophagy inducer, thus, we speculate that Sal may be a potential drug to enhance the anti-HCC sensitivity of 5-FU.

To evaluate the potential antitumor effects of Sal and 5-FU on HCC, we conducted CCK8 assays on Hepa1-6, Huh7, and HepG2 HCC cell lines to measure their cell viability after drug exposure. The determination of drug dosages was based on previous literature reports (Qin et al., 2018; Hu et al., 2020). The findings revealed that 5-FU diminished the cell viability of Hepa1-6, Huh7, and HepG2 cells in a dose-dependent manner, with the impact intensifying as the concentration of 5-FU increased (Figure 1B). Additionally, the administration of Sal in isolation also resulted in a marked reduction in the viability of HCC cells (Figure 1C). Importantly, the concurrent application of Sal and 5-FU significantly amplified the suppressive effect of 5-FU on HCC cells, exhibiting a more potent capability to inhibit cancer cell viability than when either drug was used in isolation (Figure 1D). Significantly, the co-treatment with Sal and 5-FU exerted no adverse impact on the cell viability of the normal mouse hepatocyte cell line AML12. Relative to the control group, the Sal treatment group exhibited no significant alterations in cell viability. Consistently, the co-administration of Sal and 5-FU did not lead to a significant decrease in hepatocyte viability compared to 5-FU monotherapy, suggesting that the synergistic application of Sal and 5-FU bolstered the antitumor effect without exacerbating toxicity to normal hepatocytes (Figure 1E). This effect was confirmed by monoclonal formation assays, providing evidence of their potential to work together effectively against HCC (Figure 1F). Moreover, wound healing assays indicated that the simultaneous administration of Sal with 5-FU significantly curtailed the metastatic potential of HCC cells (Figure 1G; Supplementary Figure S1D). The results indicated that 5-FU showed increased sensitivity in HCC with higher mitophagy levels, and Sal emerged as a candidate that could potentially have overcome the limitations of 5-FU therapy.

### 3.2 Salidroside increases 5-fluorouracil anti-HCC sensitivity by inducing mitophagy

**FIGURE 2:**
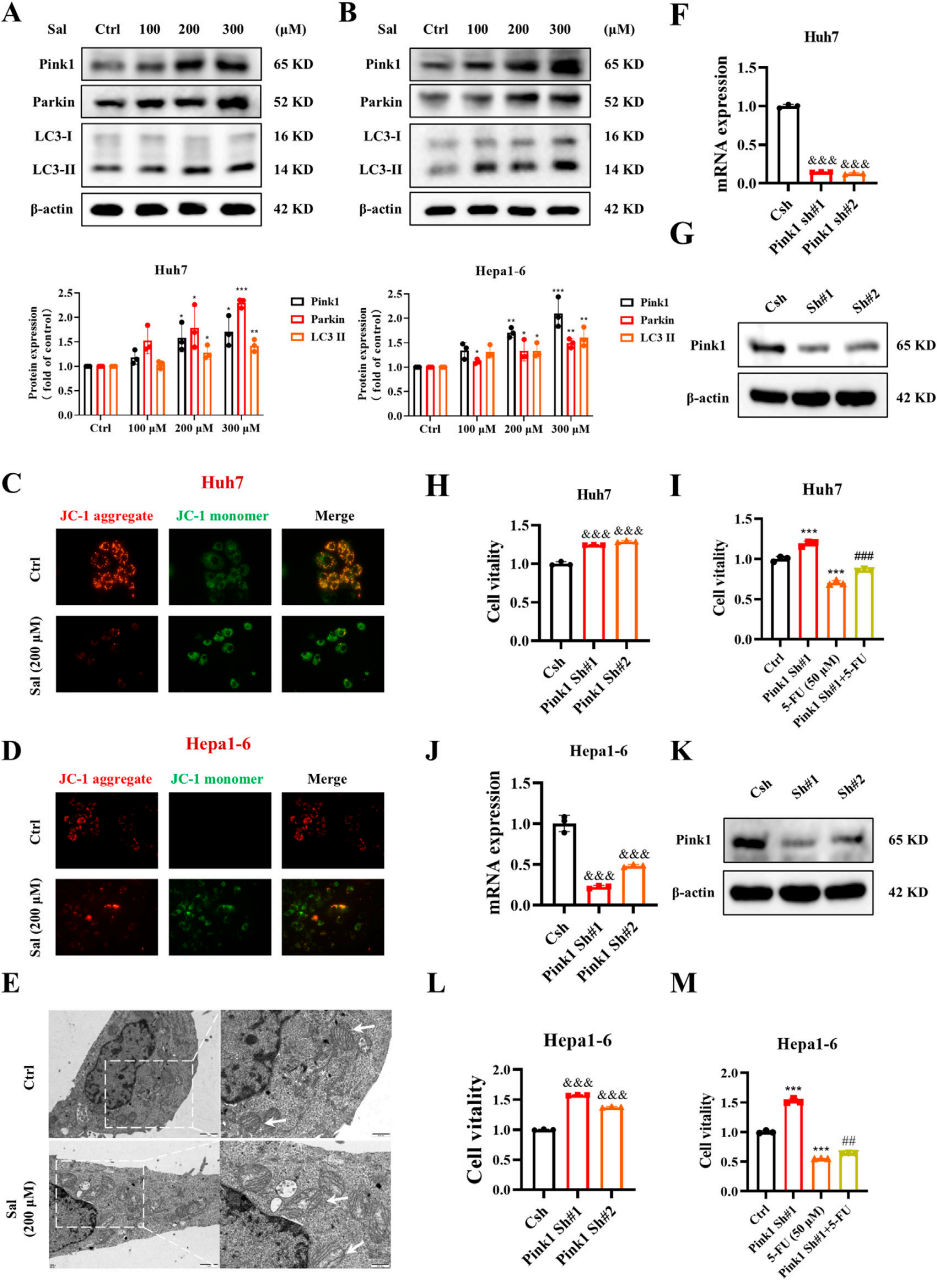
Salidroside increases 5-fluorouracil anti-HCC sensitive by inducing mitophagy. **(A, B)** The protein abundances of Pink1, Parkin and LC3 were determined after treatment with various concentrations of Sal for 24 h **(C, D)** Detection of mitochondrial membrane potential was assayed by JC-1 staining. **(E)** TEM of Huh7 cells. White arrows denote mitochondria. **(F, G)** Knockdown efficiency of Pink1 shRNA in Huh7 cells detected by qPCR and Western blot. **(H, I)** Cell viability was assayed. **(J, K)** Knockdown efficiency of Pink1 shRNA in Hepa1-6 cells detected by qPCR and Western blot. **(L, M)** Cell viability was assayed. The data are expressed as mean ± SD (n = 3); *p < 0.05, **p < 0.01, and ***p < 0.001 vs. Control solvent. ^&&&^ *p* < 0.001 vs. Csh. ^##^ *p* < 0.01, ^###^ *p* < 0.001 vs. 5-FU.

To further assess whether Sal exerts a potent anti-HCC effect with 5-FU by inducing mitophagy in HCC cells, we conducted Western blot analysis targeting key mitophagy biomarkers. The findings indicated a dose-dependent upregulation of Pink1, Parkin, and LC3-II in Huh7 and Hepa1-6 HCC cell lines upon Sal treatment, thereby implicating Sal in the activation of mitophagy in HCC cells (Figures 2A, B). Moreover, JC-1 staining revealed that Huh7 and Hepa1-6 cells exhibited stronger green fluorescence under Sal treatment, indicating a decrease in mitochondrial membrane potential, a sign of mitochondrial dysfunction (Figures 2C, D). Additionally, TEM findings depicted condensation of the mitochondrial matrix and swelling of the cristae in most mitochondria, suggesting severe mitochondrial damage (Figure 2E). Collectively, these observations proposed that Sal-induced mitophagy in HCC might have occurred before mitochondrial dysfunction, and excessive mitophagy had resulted in mitochondrial damage. Subsequently, Pink1 was targeted for knockdown using shRNA, and the efficiency of this knockdown was confirmed (Figures 2F, G). The knockdown of Pink1, recognized as a mitophagy marker in HCC cells, led to a significant enhancement in the viability of Huh7 cells (Figure 2H). Importantly, the significant knockdown of Pink1 notably reversed the inhibitory effect of 5-FU on HCC cells, an effect that was also confirmed in Hepa1-6 cells (Figures 2I–M). These findings underscored the critical role of Pink1 in the 5-FU-mediated growth inhibition of HCC cells. By inducing excessive mitophagy, Salidroside led to cellular mitochondrial dysfunction, thereby achieving a stronger anti-HCC effect than the use of 5-FU alone. These findings provided compelling evidence for the potential of Sal combined with 5-FU in the treatment of HCC, warranting further investigation into their combined use in clinical settings.

### 3.3 Sal in combination with 5-FU inhibits the development of HCC *in vivo*

**FIGURE 3:**
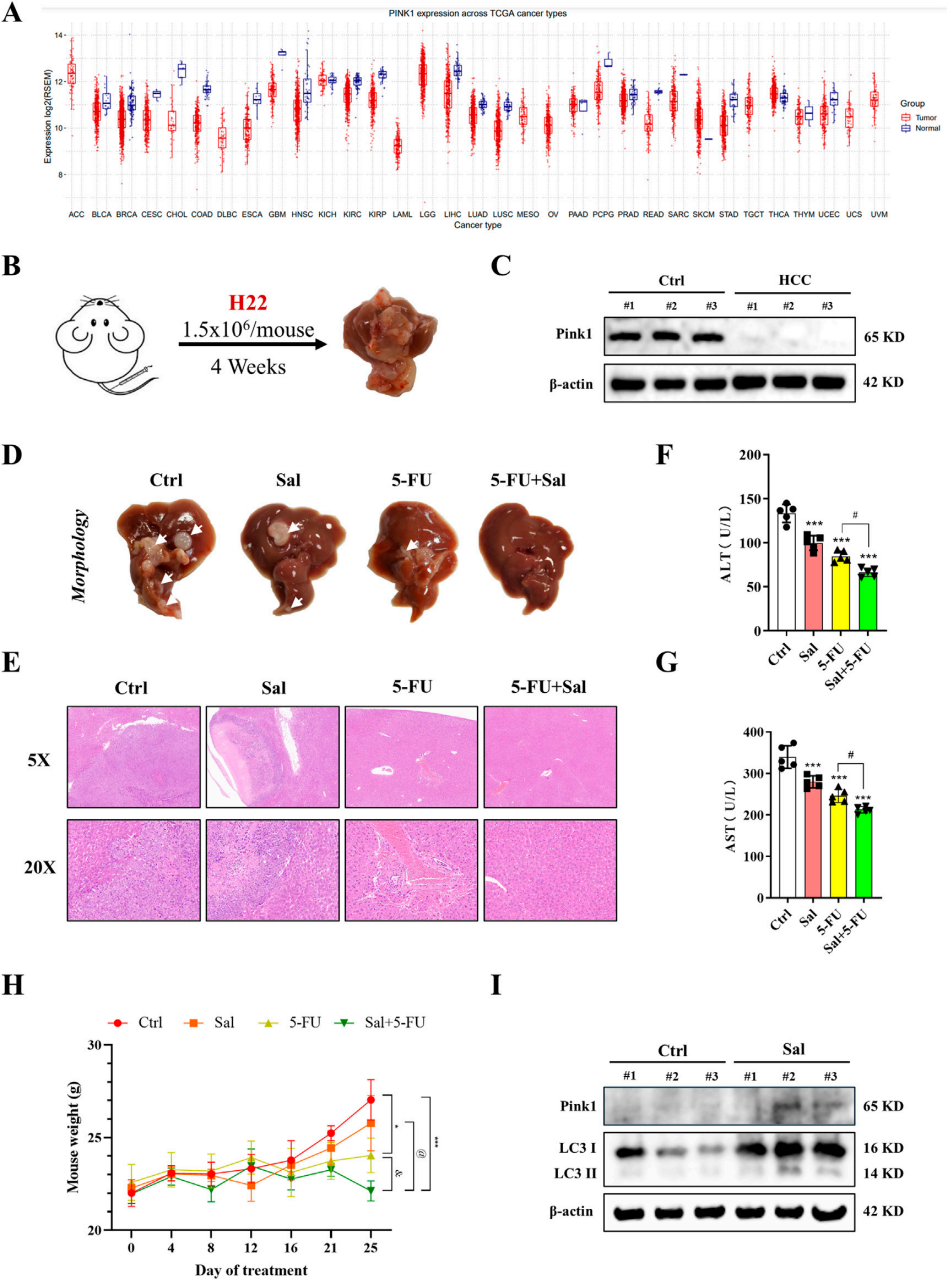
Sal in combination with 5-FU inhibits the development of HCC *in vivo*. **(A)** Pink1 expression levels in different cancer types. **(B)** Construction of primary HCC in mice. **(C)** The expression of Pink1 in carcinomas and paracarcinomas of HCC mice. **(D)** Representative images for morphological analysis of the liver. **(E)** Representative images of histopathological analysis. **(F, G)** The serum liver injury indexes were measured in mice. **(H)** The body weight changes in mice after administration of the respective drug treatments. **(I)** Changes in mitophagy-related proteins in Sal-treated mice detected by Western blot. The data are expressed as mean ± SD (n = 3); *p < 0.05, ***p < 0.001 vs. Ctrl. ^#^ *p* < 0.05 vs. 5-FU. ^@^ *p* < 0.05 vs. Sal. ^&^ *p* < 0.05 vs. 5-FU.

In the above study, Sal was identified as a modulator of mitophagy in HCC. However, this finding was initially confirmed only at the cellular level. To extend the validation, *in vivo* studies were conducted. Initially, a pan-cancer analysis revealed that Pink1 was highly expressed in patients with various types of cancer (Figure 3A). An orthotopic HCC mouse model was then established, and paraneoplastic and cancerous tissues were collected from it. The analysis confirmed the high expression of Pink1 in normal tissues, which is consistent with previous findings that Pink1 plays a protective role in HCC (Figures 3B, C). Crucially, we administered Sal in combination with 5-FU was administered to mice with HCC to investigate whether this dual therapy could further mitigate liver injury. Examination of liver morphology indicated that while control mice exhibited distinct tumours, the group treated with the drug combination showed significant improvement compared to the single-drug treatment groups (Figure 3D). The combined administration of Sal and 5-FU was found to effectively suppress the progression of HCC. Histological analysis substantiated this outcome (Figure 3E). Notably, serum liver injury markers, such as ALT and AST, demonstrated that both Sal and 5-FU could ameliorate liver injury, with the combined therapy further reducing the levels of these markers (Figures 3F, G). H22 cells, which are an ascites-type of HCC, were used to assess the impact of the drug combination on ascites formation and HCC progression in mice. The body weight of the mice indicated that the combination of Sal and 5-FU could significantly reduce ascites formation and HCC progression compared to single-agent treatments (Figure 3H). Western blot analysis provided further insights, showing that Sal significantly increased the expression of mitophagy markers Pink1 and LC3-II (Figure 3I). These results suggested that Sal induced mitophagy, thereby exhibiting stronger anti-cancer efficacy when used in combination with 5-FU compared to the use of 5-FU alone.

### 3.4 Salidroside-induced mitophagy leads to senescence and mitosis arrest in HCC

**FIGURE 4:**
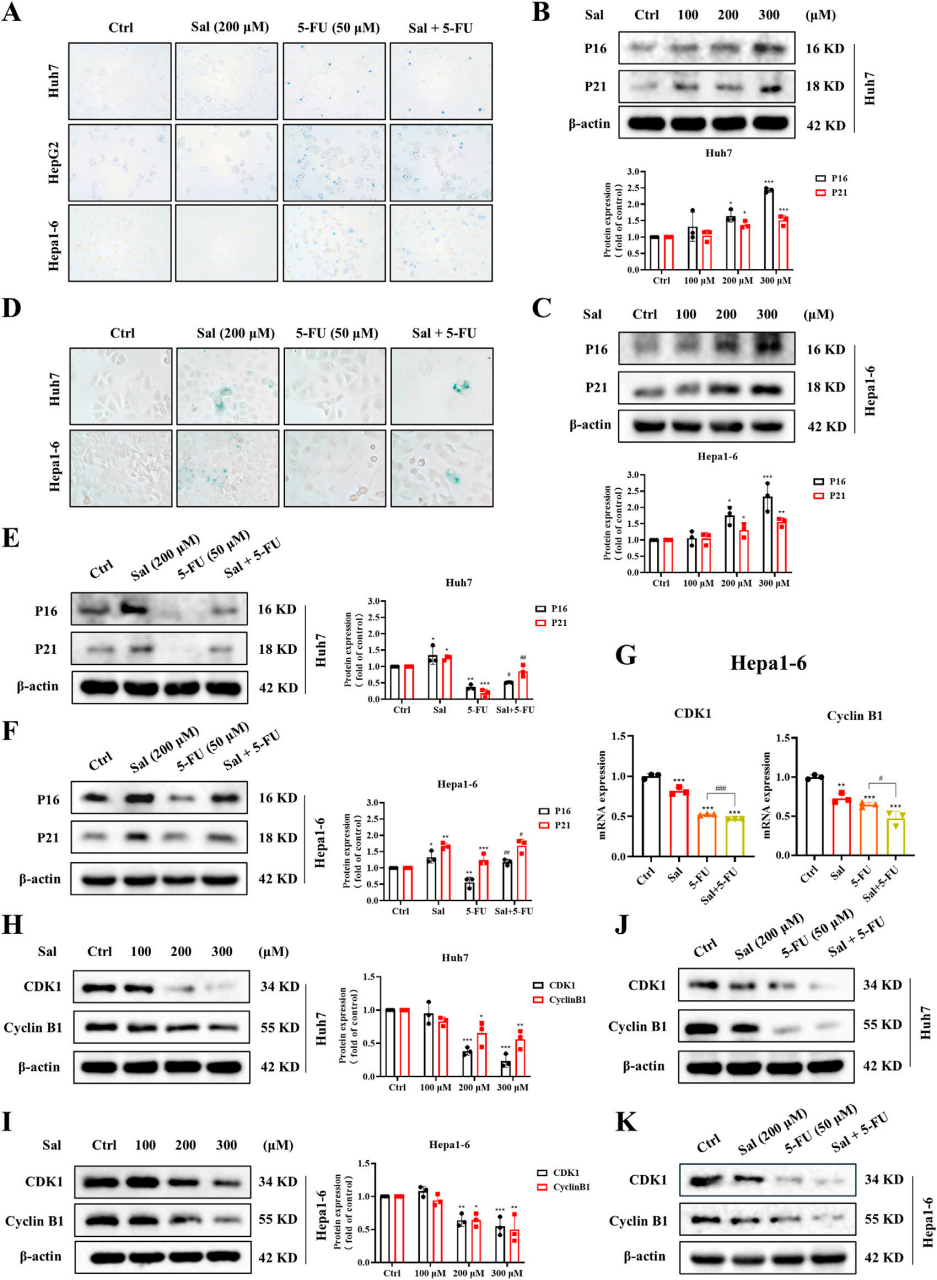
Salidroside-induced mitophagy leads to cell senescence and mitosis arrest in HCC. **(A)** The death of HCC cells was assayed by trypan blue staining. **(B, C)** Western blot was used to analyze the expression of P16 and P21 under Sal treatment. **(D)** The senescence of HCC cells was assayed by SA-β-Gal staining. **(E, F)** Changes in the expression of P16 and P21 under co-treatment of Sal and 5-FU were analyzed by Western blot. **(G)** Relative mRNA levels of CDK1 and CyclinB1 in HCC cells under the indicated drug treatments. **(H–K)** Western blot was used to analyze the expression of CDK1 and CyclinB1 in HCC cells. The data are expressed as mean ± SD (n = 3); *p < 0.05, **p < 0.01, and ***p < 0.001 vs. Control solvent. ^#^ *p* < 0.05, ^##^ *p* < 0.01, ^###^ *p* < 0.001 vs. 5-FU.

Subsequently, we embarked on a more in-depth study of the mechanisms by which Sal enhances the efficacy of 5-FU in HCC. A reduction in mitochondrial membrane potential is a pivotal event during the early stages of apoptosis (Wang et al., 2024), prompting us to initially examine whether Sal could induce HCC cell death. Trypan blue staining revealed that Sal neither facilitated HCC cell death nor augmented 5-FU-induced cell death (Figure 4A). Studies have shown that mitochondrial dysfunction is considered a causative factor in cellular senescence (Victorelli et al., 2023; Correia-Melo et al., 2016; Passos et al., 2010), and excessive mitophagy leads to this dysfunction. Furthermore, the PINK1/Parkin pathway can inhibit cellular mitosis by sequestering TBK1 in damaged mitochondria (Sarraf et al., 2019). Therefore, we hypothesised that mitophagy might be a key pathway in the regulation of cellular senescence and mitosis. Consequently, we concurrently monitored the impact of Sal on both senescence and mitosis in HCC cells, aiming to elucidate its role in the complex interplay between these processes.

Our findings revealed that Sal, in a dose-dependent manner, elevated the protein expression of senescence markers P16 and P21 (Figures 4B, C). Notably, the combination of Sal with 5-FU significantly amplified the senescence induced by 5-FU in HCC cells (Figures 4E, F). This observation was corroborated by SA-β-Gal Staining (Figure 4D). Moreover, Sal inhibited the gene and protein expression of mitotic markers CDK1 and CyclinB1 (Figures 4G–I), with an enhanced effect observed when Sal was used in conjunction with 5-FU (Figures 4G, J, K). In summary, Sal was found to potentiate the anti-HCC effects of 5-FU by inducing cellular senescence and suppressing mitosis in HCC cells.

### 3.5 Salidroside targets YIPF5 to enhance 5-fluorouracil senescence and mitosis arrest

**FIGURE 5:**
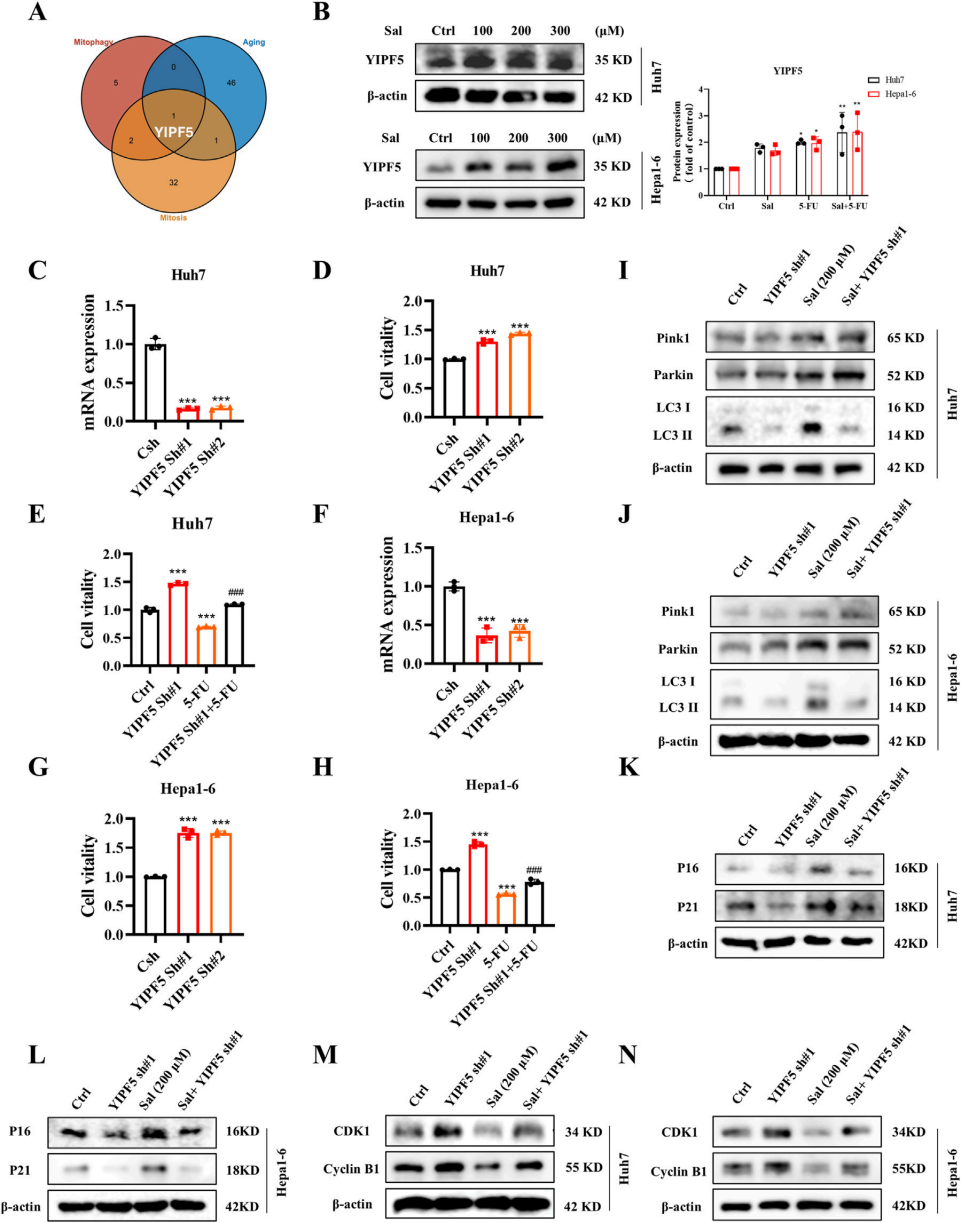
Salidroside targets YIPF5 to enhance 5-FU senescence and mitosis arrest. **(A)** A Venn diagram was drawn to show the genes with a high correlation to mitophagy, senescence and mitosis among the possible targets of Sal. **(B)** The protein abundances of YIPF5 after Sal treatment were investigated. **(C)** Knockdown efficiency of YIPF5 shRNA in Huh7 cells detected by qPCR. **(D, E)** Cell viability was assayed. **(F)** Knockdown efficiency of YIPF5 shRNA in Hepa1-6 cells detected by qPCR. **(G, H)** Cell viability was assayed. **(I, J)** The protein abundances of Pink1, Parkin and LC3 were investigated. **(K, L)** The protein abundances of P16 and P21 were investigated. **(M, N)** The protein abundances of CDK1 and CyclinB1 were investigated. The data are expressed as mean ± SD (n = 3); *p < 0.05, **p < 0.01, and ***p < 0.001 vs. Control solvent. ^###^ *p* < 0.001 vs. 5-FU.

Moving forward, the HERB database (http://herb.ac.cn/) was accessed to identify traditional Chinese medicine targets associated with Sal. Subsequently, Gene Set Variation Analysis (GSVA) was applied to score HCC samples within the TCGA dataset for mitophagy, senescence, and mitosis. A correlation analysis was then conducted with the potential targets of Sal, culminating in the identification of YIPF5, a high-scoring, Sal-specific molecular target with potential anti-HCC properties (Figure 5A).

During mitophagy, mitochondria are first encapsulated by vesicles called “barrier membranes”, which are mainly derived from the endoplasmic reticulum (ER) and the Golgi (Lin et al., 2022). YIPF5, as a multichannel membrane protein, is involved in the transport steps between the ER and the Golgi (Yoshida et al., 2008). It has been reported that YIPF5 facilitates the translocation of STING from the ER to the ER-Golgi intermediate compartment (ERGIC), which is a key step in STING activation. The ERGIC, containing STING, is a membrane source for LC3 lipidation (Ma et al., 2023). Thus, we innovatively proposed that YIPF5 might be a key target involved in Sal-regulated HCC mitophagy, which in turn affected senescence and mitosis.

Our experimental results demonstrated that YIPF5 protein levels showed a dose-dependent increase in response to Sal (Figure 5B). The knockdown of YIPF5 notably enhanced the viability of Huh7 cells, and this effect was particularly pronounced when the cells were subjected to 5-FU treatment, a phenomenon that was also observed in Hepa1-6 cells (Figures 5C–H). Notably, the knockdown of YIPF5 did not alter the expression of Pink1 and Parkin proteins (Figures 5I, J). Instead, it facilitated cellular senescence and induced mitotic arrest, potentially through its influence on the production of LC3-II (Figures 5K–N). In summary, Sal stimulated the generation of autophagosome membrane by inducing YIPF5 expression, which led to over-targeted autophagy of mitochondria, causing mitochondrial dysfunction, and thus altering the senescence and mitotic signalling pathway in HCC cells.

## 4 Discussion

The clinical application of 5-FU is often constrained by its narrow therapeutic window and pronounced toxic side effects, which are considered major challenges (Valencia-Lazcano et al., 2023). The urgent need is to identify new pharmacological strategies, and the use of combination therapies with multiple drugs is a common approach to mitigate the toxic side effects of chemotherapy and potential drug resistance (Yuan et al., 2023). FOLFOX, a combination therapy comprising folinic acid (FnA, FOL), fluorouracil (5-Fu, F), and oxaliplatin (OxP, OX), has long been considered as a standard treatment for colorectal cancer and HCC. However, the clinical application of FOLFOX is still hindered by significant challenges, including nonspecific drug delivery, high toxicity, and prolonged treatment duration (Guo et al., 2021). Interventional hepatic arterial infusion chemotherapy (HAIC) is a regional therapeutic approach that increases local drug concentrations by directly delivering chemotherapeutic agents to the arterial branches associated with the tumor. The combination of HAIC with the FOLFOX regimen (HAIC-FO) has demonstrated tolerable toxicity and promising efficacy, offering a ray of hope for patients with HCC (Li S. H. et al., 2023; Zheng et al., 2022; Lyu et al., 2022).

In our study, we fully harnessed the holistic regulatory and low toxicity advantages of traditional Chinese medicine and discovered a Chinese medicinal monomer, Sal, which has a strong anti-cancer effect when used in combination with 5-FU. Our research also honed in on the impact of Sal on the modulation of mitophagy in HCC cells, demonstrating a significant enhancement of this process. It's important to note that Sal-induced mitophagy is not solely about the degradation of impaired mitochondria-a common perception that attributes its function to the mitigation of oxidative stress in tumour cells. Mitophagy serves a dual role in tumorigenesis, and the intricate regulatory mechanisms of mitochondria in various physiopathological contexts, along with the complex interplay between different mitochondrial pathways, remain critical areas that warrant further exploration. The influence of mitophagy is contingent upon the tumour type and stage; it has been observed that mitophagy might precede mitochondrial dysfunction, and excessive mitophagy could indeed precipitate such dysfunction (Lin et al., 2023; Chen et al., 2019). Our results indicated that in Sal-treated HCC cells, there was a significant reduction in mitochondrial membrane potential, mitochondrial swelling, and disarray of the mitochondrial cristae, suggesting that Sal enhanced the anti-HCC efficacy of 5-FU by triggering excessive mitophagy, leading to mitochondrial dysfunction.

Subsequently, we delved into the relationship between the induction of mitophagy and the decline in HCC cell viability. After ruling out the possibility that Sal directly promoted HCC cell death, we discovered a correlation between the senescence of HCC cells and the effects of mitotic blockade. Cancer arises from the deregulation of the cell cycle, with normal cells proliferating only upon receiving growth-inducing signals or specific pro-division signals, a regulation that is absent in cancer cells. Cell cycle arrest is recognized as a potential therapeutic target in many malignancies and is pivotal in both the prevention and treatment of tumours.

In general terms, cells that have ceased to grow can be categorized into three main groups: quiescent cells, terminally differentiated cells, and senescent cells (Marescal and Cheeseman, 2020; Ma et al., 2019; Calcinotto et al., 2019). Senescence is distinct from the quiescent state in that it involves an irreversible halt in the cell cycle. This state of senescence is regulated by pathways dependent on p16^INK4a^ and p21^Cip1^, with senescent cells accumulating in the G1 phase of the cell cycle, as they are incapable of progressing to the S phase to initiate DNA synthesis (Herranz and Gil, 2018). Sal exerted a dual blockade on the M phase and the G1/S transition in HCC cells, thereby inducing a permanent cell cycle arrest. This mechanism may have been instrumental in significantly amplifying the anticancer sensitivity of 5-FU in HCC. By targeting key cell cycle checkpoints, the combination of Sal and 5-FU enhanced anti-cancer efficacy, presenting a promising strategy for the treatment of HCC.

## 5 Conclusion

**FIGURE 6:**
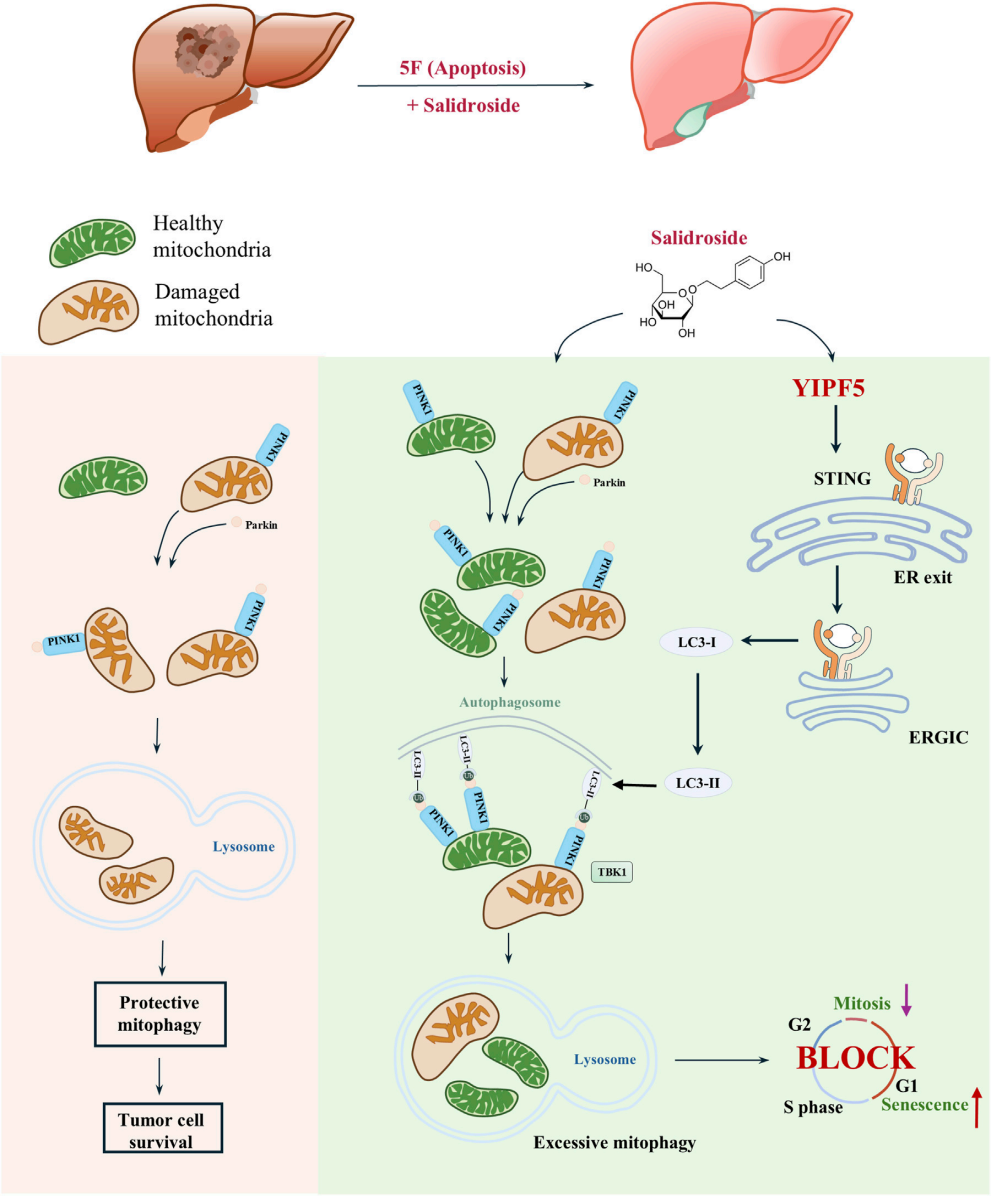
YIPF5 is required for Salidroside to enhance 5-FU anti-HCC by regulating mitophagy. In this study, Sal exhibits potent anti-cancer effects when used in combination with 5-FU, through a mechanism distinct from that of 5-FU. Sal induces mitochondrial dysfunction by triggering excessive mitophagy in HCC cells, which in turn promotes cellular senescence and mitotic arrest. Importantly, this effect was achieved by targeting YIPF5, a channel protein that plays a crucial role in STING activation. YIPF5 facilitates the transport of STING from the ER to the ERGIC, which is a key step in the activation process. The STING-containing ERGIC serves as a membrane source for LC3 lipidation, thereby inducing mitophagy and triggering a series of downstream signaling pathways.

In summary, we innovatively proposed that a high level of mitophagy could enhance the sensitivity to 5-FU. For the first time, we discovered that the natural product Sal, when used in combination with 5-FU, exhibited a potent effect against HCC. Additionally, we elucidated the regulatory role of YIPF5 in HCC, laying the groundwork for the development of targeted therapies for HCC in the future (Figure 6). We introduced a safe and effective treatment through an innovative strategy of drug repurposing, offering a promising method for effective HCC treatment.

## Data Availability

The original contributions presented in the study are included in the article/Supplementary Material, further inquiries can be directed to the corresponding author.

## Supplementary material

SUPPLEMENTARY FIGURE S1 Identification mitophagy subtypes in HCC. **(A)** Unsupervised Consensus Clustering using the k-means method with 1000 iterations to ensure clustering stability, the consensus matrix of patients in the TCGA cohort with k = 2. **(B)** Principal component analysis of mitophagy subtypes in the TCGA cohort. **(C)** Differences in ssGSVA scores for genes between the two clusters. **(D)** Wound healing assays were performed to detect Huh7 cells migration; ****P* < 0.001.

## Abbreviations

Sal, Salidroside; 5-FU, 5-Fluorouracil; HCC, hepatocellular carcinoma; ALT, alanine aminotransferase; AST, aspartate aminotransferase; FBS, fetal bovine serum; PCR, polymerase chain reaction; DMEM, Dulbecco’s modified essential medium; PBS, Phosphate-buffered saline; TEM, transmission electron microscope; H&E, Haematoxylin-eosin staining; PFA, paraformaldehyde; SA-β-Gal, Senescence associated β-galactosidase; TCGA, The Cancer Genome Atlas; PCA, Principal component analysis; OS, overall survival; DFI, disease-free interval; DSS, disease-specific survival; PFS, progression-free survival; GSVA, Gene Set Variation Analysis; ER, endoplasmic reticulum; ERGIC, ER–Golgi intermediate compartment.
