# Supplementary figures and images for: Salidroside enhances 5-fluorouracil sensitivity against hepatocellular carcinoma via YIPF5-induced mitophagy
Source: Front Pharmacol. 2025 Jan 6;15:1503490. doi: 10.3389/fphar.2024.1503490 (PMC11743563; doi:10.3389/fphar.2024.1503490)

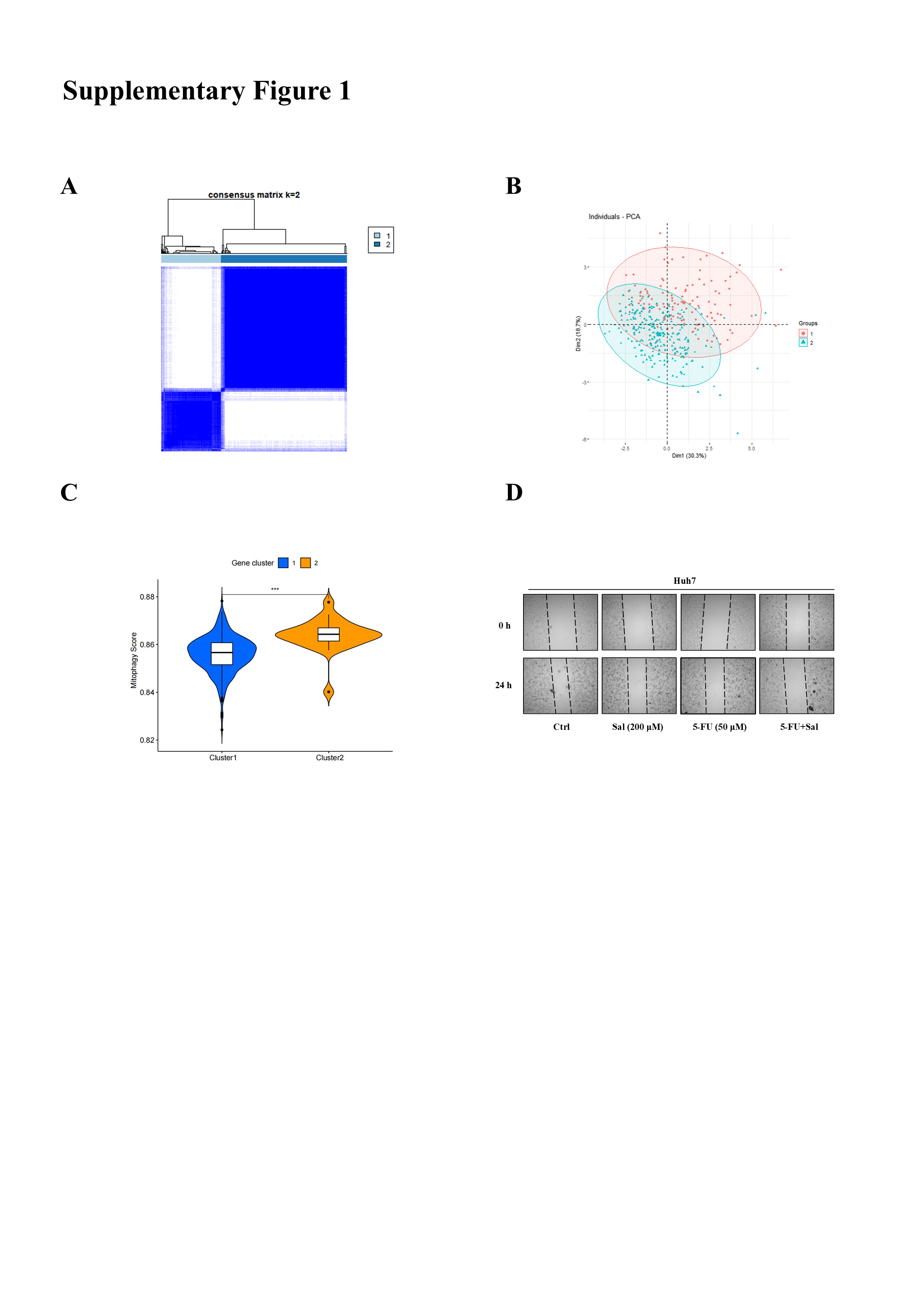

Supplement: Supplementary file 1 [file Image1.tif]
